# Algorithm for the Evaluation of Imperfections in Auto Bodywork Using Profiles from a Retroreflective Image

**DOI:** 10.3390/s140202476

**Published:** 2014-02-05

**Authors:** Ramon Barber, Valerie Zwilling, Miguel A. Salichs

**Affiliations:** 1 System Engineering and Automation Department, Carlos III University, Madrid 28911, Spain; E-Mail: salichs@ing.uc3m.es; 2 Peugeot Citroöen Automobiles SA, PSA, Velizy 78943, France; E-Mail: valerie.zwilling@mpsa.com

**Keywords:** imaging systems, diffraction, digital image processing, sensor application, image algorithm

## Abstract

Nowadays the automobile industry is becoming more and more demanding as far as quality is concerned. Within the wide variety of processes in which this quality must be ensured, those regarding the squeezing of the auto bodywork are especially important due to the fact that the quality of the resulting product is tested manually by experts, leading to inaccuracies of all types. In this paper, an algorithm is proposed for the automated evaluation of the imperfections in the sheets of the bodywork after the squeezing process. The algorithm processes the profile signals from a retroreflective image and characterizes an imperfection. It is based on a convergence criterion that follows the line of the maximum gradient of the imperfection and gives its geometrical characteristics as a result: maximum gradient, length, width, and area.

## 1. Introduction

The automobile industry is currently very concerned about the quality of the vehicles produced. One of the aspects in which significant progress is being made is the analysis of the quality of the car sheets [1,2]. This work is done under a collaboration between Carlos III University and PSA Peugeot and it is under a process of patent. The goal is to build an automatic classification and quantification system to detect the imperfections in sheets of the auto bodywork due to the squeezing process [3,4].Currently, this classification is done by the direct observation of the pieces, and the objective and novelty of this work is to try to do in an automatic and in a deterministic way. The final goal is to obtain a classification that is as similar as possible to the one obtained by visual inspection.

**Figure 1.**
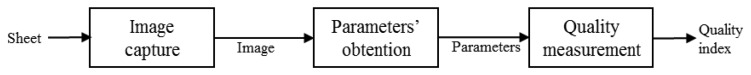
Complete process for the detection of the quality index.

The proposed algorithm uses the gradient information of multiple profiles from a retroreflective image order to characterize the defaults in an automatic way, being the main contribution of this work. The complete system that leads to the imperfection classification from the sheet is shown in Figure 1.

- Image acquisition: to establish the classification, the first step is to extract the geometrical characteristics of the sheet from an image. This image is taken by a system consisting of a motorized table, a light source with an optical fiber guide, a motorized camera, and a screen.
- Determination of the parameters: an algorithm has been implemented that allows us to extract from the images the parameters from which the geometrical properties of the sheet are determined. These parameters are related to the quality of the sheet, and the classification can be established from them.
- Determination of the quality classification: from the geometrical information obtained by the algorithm and the rules provided by the visual experience on the criticity of the imperfections, a criticity index is determined for that imperfection.

## 2. Description of the Image Acquisition System

**Figure 2.**
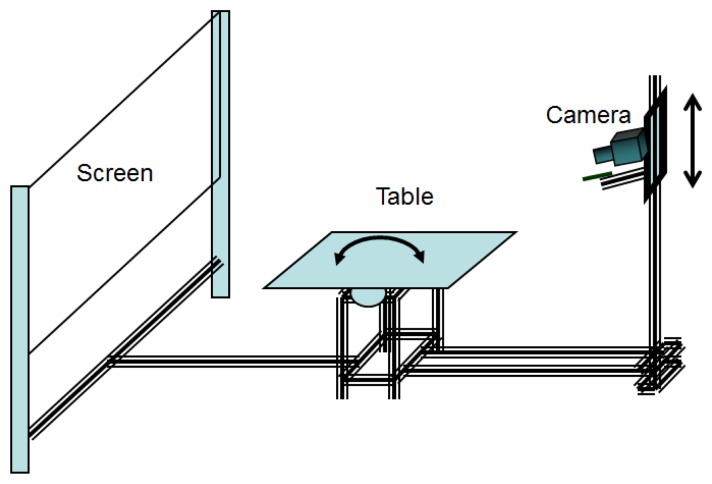
Image acquisition system.

The system for detecting imperfections in the sheets (Figure 2) consists of seven basic elements:

- A camera that captures images of the door sheets, which will be later analyzed and evaluated.
- A video conversion device that converts the analog image from the camera into a digital format so it can be processed by the PC.
- A light source: it supports the system with the adequate brightness for taking pictures. The light is guided to the optimum position and orientation through optical fiber.
- A retroreflective screen that reflects the light to the sheet and to the camera.
- A motorized table: the sheet to be analyzed is placed over it.
- A shaft driver that controls the (x, y) position of the table and the z position of the camera.
- A personal computer to control the table and the camera and to process the camera image for the performance of the classification algorithms.

The system provides adaptive but rigid joints between its components which, together with the control of the position of the table and the camera, allows us to carry out studies of repeatability in the capture and the subsequent analysis.

The image acquisition system is based on the phenomenon of light retroreflection [5,6], that enhances the existing distortions on a flat surface. This kind of method has been applied to enhance the surface variations in different fields, such as plastic materials [7,8], aerospace industry [9,10], squeezing processes in general [11], and the automobile industry in particular [5,12].

## 3. General Description of the Algorithms for the Determination of the Geometrical Parameters of the Imperfections

To characterize the imperfection, an algorithm has been developed that extracts the information from the imperfection once it is firstly selected. There are algorithms that try the surface reconstruction using least-squares techniques [13], but they do not allow the numerical quantification of imperfections of a size around 20 mm, such as the ones to be analyzed in this case. In [14] a collection of algorithms for the analysis of textures is shown, but most of them are for the classification of patterns. The work in [15] deals with a method based on the evaluation of local wall thickness and other imperfections, such us creases, using a medial axis transformation.

All the information regarding the sheet deformation is available in the profile obtained from the captured image. Taking into account that this information is the variation of the sheet deformation, the profile (signal) to work on is the derivative of the original profile. The inflection points of this signal coincide with the maxima and minima of the original profile (sheet profile). The light areas of the image coincide with the prominences and the dark ones with the valleys of the sheet. The information of the highest elevation of the imperfection is related to the value of the maximum gradient of the signal. To track the imperfection, the maximum deflection is followed. Therefore, the algorithm must search for this maximum gradient and move along the imperfection with this searching criterion.

The algorithms developed for the determination of the geometrical parameters of the imperfection and its quality index are:

- Algorithm for the obtaining of the profiles.
- Algorithm for the automated monitoring of the imperfection.
- Extraction of the parameters of the imperfection and classification.

## 4. Algorithm for the Obtaining of Profiles

### 4.1. Profile Analysis

**Figure 3.**
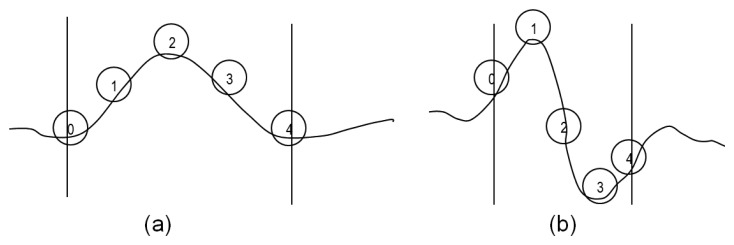
(**a**) Actual profile of the sheet; (**b**) Processed profile obtained from the image.

The first step to take is the analysis of the profile obtained from an image. The profile is typically of the form shown in Figure 3b. To get this profile, firstly a media filter is applied in the direction in which the imperfection is analyzed. Then, the resulting signal is filtered again by a filter based on wavelets.

This signal contains the information on the deformation of the sheet. Therefore, there is a correspondence between the significant points of this signal (Figure 3b) and the significant points of the sheet deformation (Figure 3a). These points are:

- Point 0: start point of the imperfection. At this point there is a change of curvature in the sheet (Figure 3a), changing the slope sign. In the processed profile (Figure 3b) it results in an inflection point.
- Point 2: point of maximum height of the defect (Figure 3a). In the processed profile (Figure 3b) it is equivalent to an inflection point at which the gradient is maximum. The information from this gradient is related to the height or severity of the imperfection.
- Point 4: end point of the imperfection. At this point there is a change of curvature in the sheet (Figure 3a), changing the slope sign. In the processed profile (Figure 3b) it results in a new inflection point.

### 4.2. Profile Obtaining

**Figure 4.**
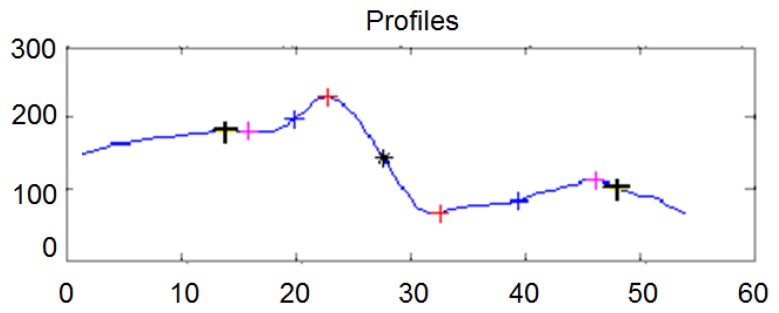
Obtaining of the ends points of the profile.

Therefore, an algorithm has been implemented that takes into account the specific characteristics of the profiles obtained from the image. This algorithm processes the profile signal, detecting the maximum, the minimum, and the inflection points and it stores the information. The steps followed by the algorithm are:

1. Image filter: for the direction in which the profile is going to be analyzed, a box is set in the image and a media filtering is performed around this direction with a width between 5 and 10 pixels. This way the effects of the illumination system are reduced.
2. Signal filter: to eliminate the noise in the signal obtained from the profile, a filter based on wavelets is applied. It is a smooth filter based on a heuristic variant of the Stein risk principle with a rescaled threshold depending on the noise level of the signal. The problem with this filter is that the filtered signal is affected by the length of the signal, especially if a big length is taken in which the initial and final ends do not contain information of the imperfection and generate a high component of noise. To avoid this problem, the signal filter divides into two stages:
  (a) In a first stage the signal is filtered by the wavelet filter and the start and end points limiting the useful information of the profile are obtained. This way, the profile size adjusts dynamically to the imperfection size in pixels. In Figure 4 the signal is cut and only the information between the start and end points is considered for the later calculations. The x axis represents the length of the imperfection in pixels, and the y axis the value of the pixel in the gray scale.
  (b) In a second stage the signal is filtered considering the information between these two ends points, applying the wavelet filter again. The result is a filtered signal independent of the length of the profile.

## 5. Algorithm for the Automated Monitoring of the Imperfection

The algorithm that extracts information from the imperfection is based on the obtaining of profiles along the imperfection, considering the ends and maximum gradient points.

The stages this algorithm is divided into are the following ones:

1. Selection of the start point for analysis of the imperfection.
2. Determination of the direction perpendicular to the imperfection at the start point by rotations around that point.
3. Progression along the imperfection determining the direction and value of the maximum gradient, as well as the ends points of the sheet deformation.
4. Completion of the algorithm.
5. Estimation of the parameters of width, length, area, and severity of the imperfection.

### 5.1. Selection of the Start Point for the Analysis of the Imperfection

**Figure 5.**
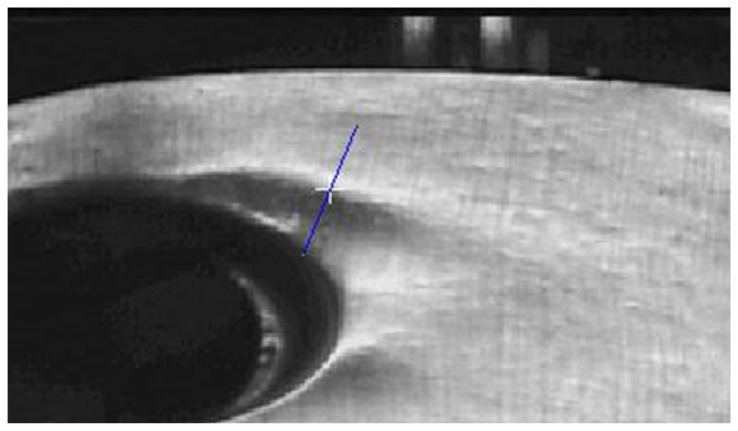
Selection of the start point of the algorithm.

The location of the imperfections are a priori known, as consequence of the squeezing process. To obtain the start point of the imperfection (Figure 5), two points must be chosen from the image: one higher and another one lower than the imperfection, the most perpendicular possible to it and in an area where the imperfection can be clearly observed.

The first profile will be automatically centered on the point of maximum gradient, corresponding with the white cross on figure (Figure 5), but keeping the selected direction. This process has to be done in the calibration process of the system for each imperfection. The following stage automatically detects the maximum gradient and the imperfection direction but it needs the initial line be close to the perpendicular, less than 45 degrees.

### 5.2. Determination of the Direction Perpendicular to the Imperfection

**Figure 6.**
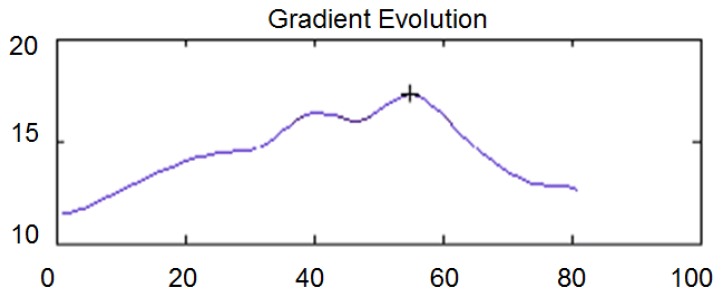
Evolution of the gradient along a rotation.

To obtain the direction perpendicular to the imperfection, where the gradient is maximum, a rotation of between −angle and +angle around that point is carried out. The evolution of the gradient along the rotation is also filtered by a signal filter based on wavelets, similar to that used for the profile filtering. Figure 6 shows the evolution of the gradient for a rotation after the application of the filter. In the x axis the value 0 corresponds to an angle of −40 and the value 80 corresponds to an angle of +40. The y axis shows the value of the gradient in gray scale per pixel.

### 5.3. Progression along the Imperfection

Once the start point and an initial direction are chosen, a recursive algorithm is used to track the imperfection. First, it follows the imperfection from the start point to the left, and then from the start point to the right. The maximum gradient criterion is followed to move along the imperfection.

The stages of this algorithm are:

- Move *n* pixels.
- Center on the point of maximum gradient.
- Perform rotations around that point between −*r* and +*r*.
- Center on the point and direction in which the gradient is maximum.
- Repeat the process iteratively until the maximum gradient point and the maximum gradient direction coincide with the previous values. In this case, a new point and a new direction of maximum gradient have been found. The ends points of the imperfection and the value of maximum gradient are stored.
- If the algorithm reaches again the same point in the following iteration and finds a local minimum, or keeps oscillating between two positions, the parameters estimated in the first iteration are then taken to continue the execution of the algorithm.
- Move other n pixels and repeat the process.

**Figure 7.**
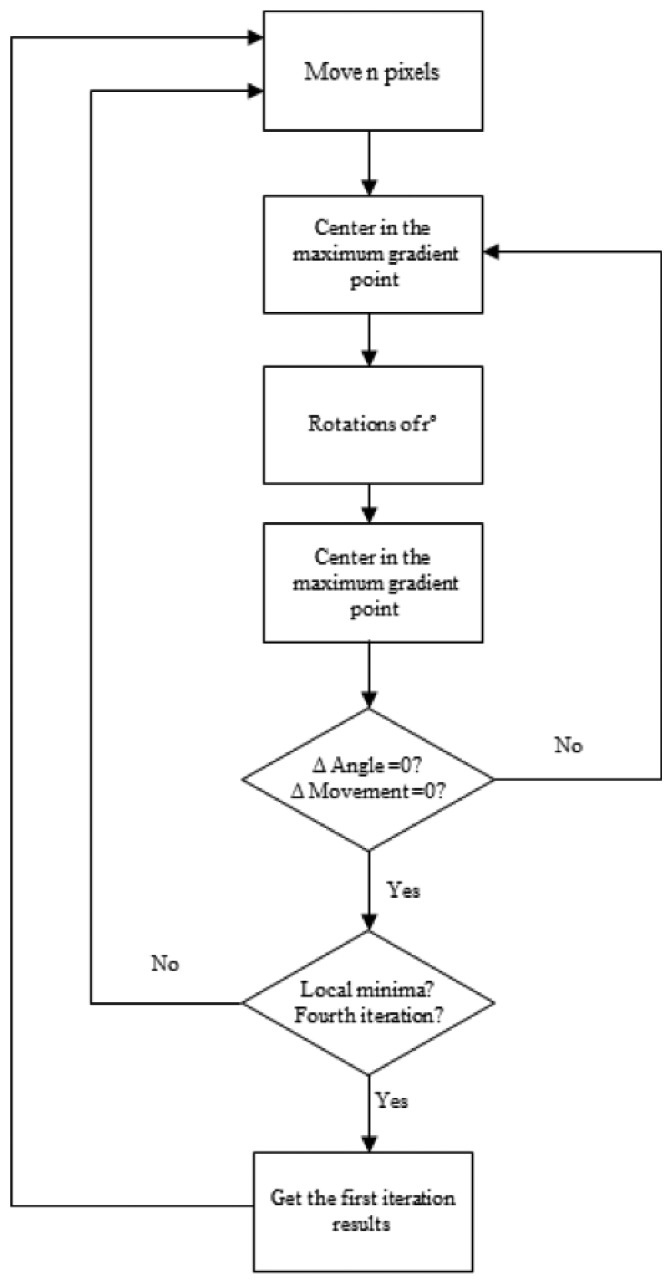
Flow chart of the algorithm.

**Figure 8.**
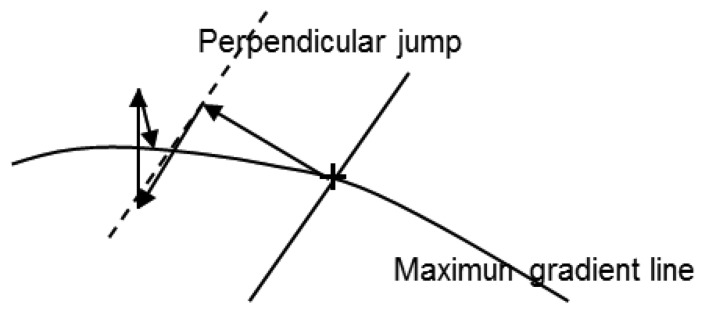
Convergence process towards a point of maximum gradient.

Figure 7 shows the flow chart of the algorithm and Figure 8 the convergence process towards a point of maximum gradient.

### 5.4. Completion of the Algorithm

The algorithm execution can end for two reasons:

1. The maximum gradient is less than a given threshold, from which it is considered that there is no longer imperfection. This value can be obtained by calculating the slope in areas where the sheet is defect-free. Its value ranges between three and five units of gray variation per pixel and it is experimentally estimated in the calibration of the image system and it depends on the curvature of the metal sheet.
2. While moving along the imperfection, the algorithm reaches an area where the average value in the gray scale is too high or too low. It happens, for example, when the imperfection reaches the physical limit of the sheet, as the case of the area of the door handle (low values in the gray scale) or the case of edges perpendicular to the imperfection that are a consequence of the design of the door (high values in the gray scale).

## 6. Estimation of the Geometrical Parameters of the Sheet

The parameters obtained previously can be given in pixels or millimeters. To convert pixels into millimeters, a calibration of the system is required.

From the point of maximum gradient and ends points, the calculation of the geometrical parameters of the imperfection is carried out:

- Maximum gradient: it is the highest value of all the gradients. It indicates the severity of the imperfection.
- Direction of the maximum gradient: it indicates the direction in which the imperfection is the most visible.
- Imperfection line: it is formed by the union of the points of maximum gradient and it is previously filtered by splines. This line covers the most prominent area of the imperfection, showing its length visually.
- Length of the imperfection: it is determined by the addition of the distances between the points of maximum gradient.
- Width of the imperfection: it is the longest distance between the start and end points of each profile.
- Area of the imperfection: it is calculated by integrating the area formed by the curve that joins the start and end points of each profile, previously filtered by splines.

**Figure 9.**
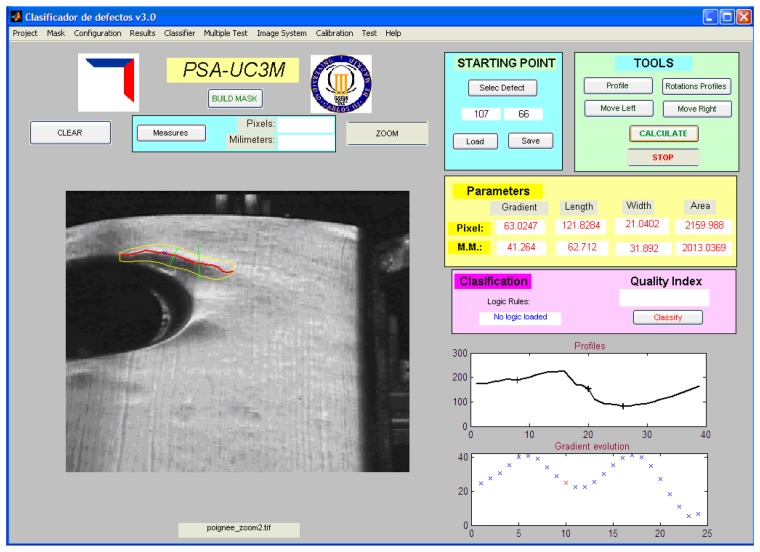
Graphical and numerical results obtained from the algorithms.

Figure 9 shows the graphical and numerical results obtained from these algorithms.

## 7. Experimental Results

For the validation of the algorithm, different tests have been carried out to various imperfections in different sheets coming from the squeezing process. Specifically, the imperfections located around the handle zone of the doors are analyzed, contrasting the results with the values estimated by experts and checking the robustness of the algorithm proposed.

### 7.1. Results from the Characterization of Several Imperfections

**Figure 10.**
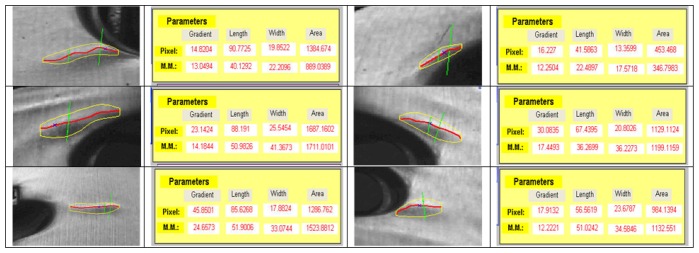
Evaluation of different imperfections.

To verify the proper performance of the algorithm, different imperfections have been analyzed (Figure 10) at different positions in different doors. Results were validate by expert staff.

### 7.2. Study of Repeatability

**Figure 11.**
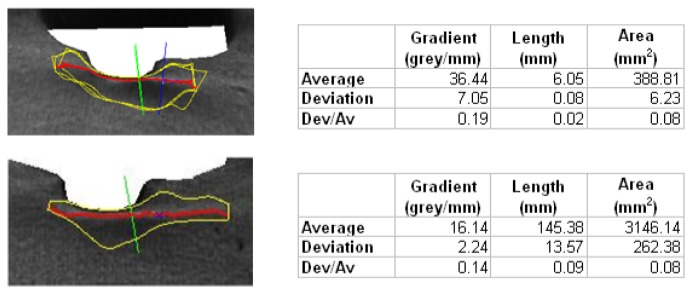
Average values and deviations for different imperfections.

Finally, to evaluate the robustness and repeatability of the algorithm, an imperfection is analyzed beginning with different start points, calculating the average, the deviation, and the weighted deviation considering 10 start points. Table 1 shows the statistics obtained in this case:

Repeating the study for other imperfections similar results are obtained, as observed in Figure 11.

## 8. Conclusions

This paper has presented an algorithm that allows us to characterize geometrically an imperfection in the auto bodywork from an image taken by retroreflection. The algorithm is based on the extraction and filtering of profiles, obtaining the point of maximum gradient, related to the height of the imperfection, and the points that delimit the width of the imperfection. From these points, the severity of the imperfection is determined, together with its width, length, and area. The experimental results obtained with this algorithm have been also validated by experts on this subject and considered for their later classification. The studies of repeatability show acceptable deviations that can be absorbed by an imperfection classification system based on fuzzy logic. Therefore, this technique can be taken as the base for establishing new quality criteria in the automobile industry.

## Figures and Tables

**Table 1.** Statistics.

	**Gradient (grey/mm)**	**Length(mm)**	**Area (*mm*^2^)**
**Average**	14.90	150.44	5396.40
**Deviation**	2.70	12.50	7774.57
**Dev/Av**	0.18	0.08	0.14
